# The Course of Neurocognitive Functioning and Prediction of Behavioral Outcome of ADHD Affected and Unaffected Siblings

**DOI:** 10.1007/s10802-018-0449-z

**Published:** 2018-08-06

**Authors:** M. van Lieshout, M. Luman, L. J. S. Schweren, J. W. R. Twisk, S. V. Faraone, D. J. Heslenfeld, C. A. Hartman, P. J. Hoekstra, B. Franke, J. K. Buitelaar, N. N. J. Rommelse, J. Oosterlaan

**Affiliations:** 1 Clinical Neuropsychology Section, Vrije Universiteit Amsterdam, Van der Boechorststraat 1, 1081 BT Amsterdam, The Netherlands; 2 Department of Psychiatry, University of Groningen, University Medical Center Groningen, Groningen, the Netherlands; 3 Department of Health Sciences, Vrije Universiteit Amsterdam, Amsterdam, the Netherlands; 4 Department of Epidemiology and Biostatistics, Vrije Universiteit Medical Center, Amsterdam, the Netherlands; 5 Departments of Psychiatry and of Neuroscience and Physiology, SUNY Upstate Medical University, Syracuse, NY USA; 6 KG Jebsen Centre for Research for Neuropsychiatric Disorders, University of Bergen, Bergen, Norway; 7 Department of Psychiatry, Donders Institute for Brain, Cognition and Behaviour, Radboud University Medical Center, Nijmegen, the Netherlands; 8 Department of Human Genetics, Radboud University Medical Center, Nijmegen, the Netherlands; 9 Department of Cognitive Neuroscience, Donders Institute for Brain, Cognition and Behaviour, Radboud University Medical Center, Nijmegen, the Netherlands; 10 Karakter Child and Adolescent Psychiatry University Center, Nijmegen, the Netherlands

**Keywords:** ADHD, Neurocognitive functioning, Course, Symptom severity, Overall functioning

## Abstract

Longitudinal studies on the course of neurocognitive functioning of children with ADHD and their unaffected siblings are scarce. Also, it is unclear to what extent that course is related to ADHD outcomes. A carefully phenotyped large sample of 838 Caucasian participants (ADHD-combined type: *n* = 339, unaffected siblings: *n* = 271, controls: *n* = 228; mean age at baseline = 11.4 years, mean age at follow-up = 17.3 years, *SD* = 3.2) was used to investigate differences in the course of neurocognitive functioning of ADHD affected and unaffected siblings versus controls, and to investigate the relationship between neurocognitive change and ADHD outcomes. At baseline, an aggregated measure of overall neurocognitive functioning and eight neurocognitive measures of working memory, timing (speed/variability), motor control, and intelligence were investigated. Outcomes at follow-up were dimensional measures of ADHD symptom severity and the Kiddie-Global Assessment Scale (K-GAS) for overall functioning. At follow up, affected and unaffected siblings trended to, or fully caught up with performance levels of controls on four (44.4%) and five (55.6%) of the nine dependent variables, respectively. In contrast, performance in remaining key neurocognitive measures (i.e. verbal working memory, variability in responding) remained impaired at follow-up. Change in neurocognitive functioning was not related to ADHD outcomes. Our results question the etiological link between neurocognitive deficits and ADHD outcomes in adolescents and young adults.

**Electronic supplementary material:**

The online version of this article (10.1007/s10802-018-0449-z) contains supplementary material, which is available to authorized users.

Attention-Deficit/Hyperactivity Disorder (ADHD) is characterized by impairing symptoms of inattention and/or hyperactivity/impulsivity (American Psychiatric Association 2013) that are to a large extent persistent into adolescence and young adulthood (Franke et al. 2012; Greydanus et al. 2007; van Lieshout et al. 2016). Although neurocognitive dysfunctions are at the heart of the majority of models on ADHD (Barkley 1997; Pennington and Ozonoff 1996; Sergeant 2000; Rapport et al. 2009; Sonuga-Barke et al. 2010), little is known about the longitudinal course of these dysfunctions and its relation to behavioral outcomes in ADHD.

Different perspectives exist on the course of neurocognitive functions and its relation with behavioral outcomes of ADHD in particular. The first perspective is that of ADHD reflecting a “maturational lag”, focusing on the course of neurocognitive functions, but not its relation with behavioral outcomes: Children with ADHD will remit from their impairments in neurocognitive functioning and their ADHD symptoms during development and catch-up with normative development (Berger et al. 2013; Drechsler et al. 2005; Klein and Mannuzza 1991; Shaw et al. 2007, 2012). Following the maturational lag hypothesis, a partial or full catch-up of neurocognitive functioning to the level of controls is expected for all neurocognitive functions.

From the following models, hypotheses regarding the relationship between neurocognitive functioning (over time) and (ADHD) behavior are derived. Importantly, these models are not necessarily discrete; overlap might exist. One important model is the endophenotype model. This model describes the relationship between neurocognitive functioning and phenotypic characteristics, by stating that neurocognitive functions mediate between the genetic liability for the disorder and the phenotypic expression (Gottesman and Gould 2003). Although this model not explicitly zooms in on longitudinal aspects, according to this model, it is likely to expect that improvements in neurocognitive functioning relate to better behavioral outcomes and that deterioration of neurocognitive functioning would relate to worse behavioral outcomes. An extension is postulated by Halperin and Schulz (2006), who specifically focused on longitudinal aspects and differentiated between different types of neurocognitive functions. They hypothesize that the normalization of functions requiring high mental effort and/or conscious control may underlie symptom improvement, while impairment in functions requiring lower levels of effort and/or control may be persistent, core deficits in ADHD, unrelated to symptom recovery (Halperin and Schulz 2006). Following this model it is thus expected that strong higher order neurocognitive functions are related to better ADHD outcomes, while (some, not necessarily all) lower order neurocognitive functions remain impaired and are not related to ADHD outcomes. A contrasting model is that neurocognitive dysfunctions have no etiological role in ADHD. They can at best be seen as some type of comorbid condition, related to the same underpinnings as ADHD symptoms, but not necessarily causally related. Presence of co-occurring neurocognitive problems then may mark a more severe form of the disorder as neurocognitive deficits and symptoms may independently contribute to impairment (Coghill et al. 2014a; van der Meer et al. 2013). According to this model, longitudinal change in neurocognitive functioning is causally unrelated to ADHD outcomes. An extension to this model is a phenomenon called Berkson’s bias (Peritz 1984): possibly, only children with ADHD symptoms *and* neurocognitive dysfunctions are clinically referred (because they are more severely impaired), while children with symptoms *without* neurocognitive dysfunctions may be ‘missed’. This may lead to a distorted view on the role of neurocognitive dysfunctioning in ADHD. A case of Berkson’s bias should thus be considered when there is a longitudinal relationship between neurocognitive functioning and overall functioning. In summary, there is an ongoing debate on the role of (the longitudinal course of) neurocognitive functioning in the emergence and further course of ADHD.

Both cross-sectional and longitudinal studies have investigated the relationship between neurocognitive functioning and ADHD. Although a longitudinal approach can provide us with more insight into the possible causal role of neurocognitive functioning for ADHD outcome, studies using such a design are in a minority. Cross-sectional studies performed so far suggest that at least three major domains seem to play a key role in ADHD: cognitive control, reward processing, and temporal processing (Castellanos and Tannock 2002; Durston et al. 2011; Sonuga-Barke et al. 2010; Wahlstedt et al. 2009), although, for example, also processing speed and motor control appear relevant (Faraone et al. 2015). Longitudinal studies investigating possible causal relations between neurocognitive functioning and ADHD outcome show heterogeneous results: Several longitudinal studies that looked into the predictive value of neurocognitive deficits in preschool children showed that early neurocognitive deficits predicted the onset of ADHD in (younger) childhood (Pauli-Pott and Becker 2011; Rajendran et al. 2013a, b; van Lieshout et al. 2013) or ADHD symptoms in adolescence (Sjöwall et al. 2015). For example, it was recently demonstrated that neurocognitive deficits at age 3–4 years had an overall predictive power of 67% for the emergence of ADHD at age 6 years in children with behavioral problems (e.g. high activity level, defiance, aggression or impulse control (Breaux et al. 2016). In addition, some studies investigated the course of neurocognitive functioning in relation to ADHD outcomes and found support for earlier mentioned theories in which neurocognitive improvement was related to better ADHD outcomes (Biederman et al. 2009; Coghill et al. 2014a, b; Michelini et al. 2016; Miller et al. 2013; Rajendran et al. 2013a, b). However, several (other) studies (also) indicated limited predictive value of the course of neurocognitive functions from childhood to adolescence/young adulthood for ADHD outcomes; in these studies, the course of neurocognitive functioning was (largely) independent of the course of diagnostic status or symptoms (Coghill et al. 2014a, b; McAuley et al. 2014; van Lieshout et al. 2013). Taken together, existing studies show little convergence on the exact relationship between the course of neurocognitive functioning and ADHD outcomes in adolescence/young adulthood and findings do not suggest that there is evidence of specific neurocognitive functions that relate more strongly than others to ADHD symptom development (onset or persistence).

The inconsistent findings of previous studies examining longitudinal relationships between neurocognitive functions and ADHD may be related to methodological shortcomings (see for systematic review: van Lieshout et al. 2013). A first general issue is that sample sizes were often quite small, which may have led to statistical power problems, more specifically an increased risk of type II errors; failing to detect an effect that actually is present. Second, some aspects hindered clear interpretation regarding the role of neurocognitive change for ADHD outcomes: (a) only few studies actually did include neurocognitive measurements at two time points or more. (b) Studies mainly focused only on one specific aspect of neurocognitive functioning, while it is likely that multiple domains may be involved in the relationship between neurocognitive functioning and ADHD outcomes. A third limitation relates to the type of outcome measures: (a) most of the studies focused on diagnostic outcomes, rather than on (more sensitive) continuous measures of symptom severity (Willcutt et al. 2012; Lahey and Willcutt 2010), which may have led to an underestimation of apparent relationships between neurocognitive functioning and behavioral problems. (b) Few studies targeted outcomes other than ADHD, such as overall functioning, covering aspects of social, psychological, and academic functioning. Such outcomes may clinically be more relevant. Fourth, some issues relate to the investigation of potential confounding or moderating effects: (a) most of the studies so far did not investigate possible moderating effects of age in samples with a large age range. Investigating age is highly relevant given the ongoing neurocognitive and behavioral development from childhood into adulthood, for which some functions show a sharp transition in adolescence (Geier 2013). Further, the course of neurocognitive functioning over age may even show a non-linear pattern (Vaughn et al. 2011). (b) Few studies took effects of medication history into account, which is of importance, as pharmacological treatment may impact behavioral outcomes (Faraone and Buitelaar 2010) as well as neurocognitive functioning (Coghill et al. 2014a, b). Taken together, many limitations of previous studies may explain inconsistencies in findings currently available.

An important group in understanding the course of ADHD is the group of unaffected siblings of children with ADHD. Because affected siblings share on average one-half of their genetic variants and several environmental risk factors with their unaffected sibling (some of whom have subclinical levels of ADHD symptoms), these unaffected siblings may be at-risk for developing a full diagnosis. However, in unaffected siblings, developmental outcomes can be studied independent of an ADHD diagnosis and treatment for ADHD at study entry. To our knowledge, unaffected siblings have not yet been studied longitudinally in relation to neurocognitive functioning. Cross-sectional studies of neurocognitive functioning in unaffected siblings showed mixed results: Unaffected siblings showing worse performance than controls (Rommelse et al. 2007a), with unaffected siblings not being different from their ADHD siblings (Bidwell et al. 2007; Pironti et al. 2014), or showing scores in between affected siblings and controls (Rommelse et al. 2008a, b), or showing subtle or even no deficits, while their affected siblings were impaired (Doyle et al. 2005; Fliers et al. 2010; Rommelse et al. 2008b, 2007a; Seidman et al. 2000).

The current study improves upon shortcomings of earlier studies, by prospectively studying a large sample of extensively phenotyped ADHD affected and unaffected siblings, and controls (*N* = 838). We investigated the neurocognitive course of these three groups of children to achieve two aims. First, we investigated the neurocognitive course in multiple domains (an aggregated measure of neurocognitive functioning, working memory, timing, variability, baseline speed, motor control, and IQ) comparing ADHD affected and unaffected siblings with controls. Participants were between 5 and 19 years old at baseline and re-assessed on average six years later when they were between 11 and 25 years old. Second, we mapped the course of neurocognitive functioning in multiple domains onto dimensional ADHD outcomes (symptoms and functional outcome) at follow-up, over and above baseline ADHD severity, carefully taking into account the effects of age. As several theoretical models exist on the course of neurocognitive functioning and its relationship with ADHD outcomes, and these models formulate contrasting ideas, we did not formulate hypotheses. Rather, we discussed which model(s) best fitted our results.

## Method

### Participants

A sample of 838 participants with ADHD combined type (ADHD/C; affected siblings), their unaffected siblings, and controls, aged 5 to 19 years at baseline, participated in this study. The sample was part of a follow-up study of the Dutch branch of the International Multicenter ADHD Genetics (IMAGE) study (von Rhein et al. 2015). The original sample (*N* = 1092) was contacted and invited for follow-up on average 5.9 years (*SD* = 0.8) after enrolment; 76.7% (*N* = 838) was retained successfully. Attrition analyses are described in Supplement 1.

Selection and diagnostic procedures at baseline (Müller et al. 2011) and at follow-up (von Rhein et al. 2015) have been detailed previously. Briefly, inclusion criteria for entry at baseline were an age of 5–19 years, Caucasian descent, IQ ≥ 70, no diagnosis of autism, epilepsy, general learning difficulties, brain disorders, and known genetic disorders. Inclusion criteria for the ADHD group were a (suspected) clinical diagnosis of ADHD/C as established by a registered health care professional confirmed using an extensive assessment protocol at baseline. Please see Supplement 1 for a more detailed description on selection and diagnostic procedures, as well as additional exclusion criteria regarding the data quality check. At baseline, all participants diagnosed with ADHD/C had at least six symptoms in both the inattention and hyperactive/impulsive domains endorsed on the Parental Account of Children’s Symptoms (PACS; Taylor 1986) in combination with a teacher rating and additional check of criteria such as impairment and pervasiveness. Definition of affected and unaffected siblings refers to the diagnostic status at baseline. See Supplement 1 for further details on participant inclusion. The 838 participants came from 398 different families. Included were 339 participants with ADHD/C (mean age at baseline = 11.4 years, *SD* = 2.8*;* range: 5.4–18.0, and mean age at follow-up = 17.5 years, 82.0% males); 271 unaffected siblings (mean age at baseline = 11.2 years, *SD* = 3.6*;* range: 5.2–18.5, and mean age at follow-up = 17.3 years; 41.3% males), and 228 controls (mean age at baseline = 11.6 years, *SD* = 3.2; range: 5.2–19.0, and mean age at follow-up = 16.8 years; 39.9% males).

### Measures

#### Neurocognitive Variables

Neurocognitive variables were identically measured at baseline and at follow-up. Measures were chosen at the time of baseline assessment based on their potential to discriminate between ADHD and control and in addition, their potential to act as endophenotype (e.g. associated with unaffected siblings). Included were verbal working memory (Rommelse et al. 2008a), temporal processing (time production, time reproduction, time production variability, and reaction time variability; Rommelse et al. 2008b, 2007a; Tamm et al. 2012; Toplak et al. 2006; Willcutt et al. 2012), reaction time speed (Rommelse et al. 2008b; Willcutt et al. 2012), motor control (Carte et al. 1996; Pitcher et al. 2002; Rommelse et al. 2007b), and intelligence (Frazier et al. 2004; Rommelse et al. 2008a; Willcutt et al. 2012). Although we have included measures for inhibition and visuo-spatial working memory at baseline and follow-up, we could not include these measures in the current manuscript, given that these measures were adjusted for use in the MRI scanner at follow-up. For an index of overall neurocognitive functioning an aggregated score including all neurocognitive measures described above was used. All variables were standardized into z-scores, by pooling data for the two time points and three groups, except for the variable total IQ, which was already expressed in age-adjusted normalized scores. See for further details on paradigms that were used Supplement 1 and Supplemental Table 1.

#### Outcome Measures

ADHD symptom severity at follow-up was our main dependent variable assessed as the raw score on the Conners’ Parent Rating Scale–Revised: Long version (CPRS-R:L; Conners et al. 1998) scale N, hereafter referred to as ‘current ADHD symptoms’. Scores on the Conners ADHD subscales represent combined measures of the number and severity of symptoms. The Global Assessment Scale-score (K-GAS) of the Dutch version of the Schedule for Affective Disorders and Schizophrenia for School-Age Children - Present and Lifetime Version (K-SADS; Kaufman et al. 1997) administered at follow-up to both the parent and the child ≥12 years separately, was used to measure current overall functioning. As part of the K-SADS interview, the interviewer rated psychological, social and academic functioning, resulting in an overall measure of the current level of functioning ranging between 1 (worst possible level of functioning) and 9 (best possible level of functioning) (please see Schorre and Vandvik 2004 for similar scoring systems). Interviewers from the participating centers (clinicians - i.e. child psychiatrists, psychologists-, or researchers - having a minimal degree of MSc) underwent comprehensive training by a team under the supervision of JB at the Donders Institute for Brain, Cognition and Behavior, Radboud University Medical Center, Nijmegen. Trained interviewers used the same training and supervision procedures for additional interviewers at the participating centers. Inter-rater agreement on diagnoses (K-SADS) was 0.94 (Cohen’s kappa, ADHD; von Rhein et al. 2015).

#### Covariates

Follow-up interval was defined as the time between baseline and follow-up measurement (in years). Baseline ADHD symptom severity was measured by scale N of the CPRS-R:L, and impairment at baseline was measured by the impairment scale of the Strengths and Difficulties Questionnaires (SDQ; Goodman, 1997), reported by parents (range 0–21). Pharmacological treatment of ADHD was defined as the cumulative intake of psychostimulants from age of onset until follow-up. Information on cumulative intake (mean daily dose multiplied by treatment duration corrected for age (treatment duration in months divided by [age minus the minimum start-age within the sample, i.e., 28 months])) was derived from pharmacy transcripts, and when pharmacy transcripts did not fully cover the self-reported treatment period, medication parameters of the missing period(s) were calculated from questionnaire data and were added to the measures derived from the pharmacy, see for a full description (van Lieshout et al. 2016). Age was measured as age at follow-up, in years. Sex and study site (Amsterdam/Nijmegen) were also included as covariates. See for a description of predictor and outcome variables Table 1.

**Table 1:** Descriptives of predictor and outcome variables

	ADHD		Baseline unaffected		Control		ADHD		Follow-up unaffected		Control	
	*M*	*SD*	*M*	*SD*	*M*	*SD*	*M*	*SD*	*M*	*SD*	*M*	*SD*
Age (*yrs*)	11.44	2.77	11.21	3.57	11.60	3.22	17.51	2.80	17.30	3.62	16.85	3.22
Sex (*N,* % male)	278	82.0%	112	41.3%	91	39.9%	–	–	–	–	–	–
Follow-up interval	–	–	–	–	–	–	6.07	0.64	6.09	0.61	5.25	0.72
Pharmacological treatment until follow-up (cumulative intake)	–	–	–	–	–	–	126.96	121.08	21.75	65.91	0.10	1.31
SDQ impairment (parent)	12.40	3.88	3.02	4.48	n.a.	n.a.	–	–	–	–	–	–
CPRS-R:L total symptom severity (scale N)	35.64	8.59	8.52	8.92	4.67	4.42	23.21	11.42	7.71	8.82	4.02	4.80
K-GAS-score	n.a.	n.a.	n.a.	n.a.	n.a.	n.a.	6.42	1.13	7.93	1.19	8.52	1.16
Overall measure of neurocognitive functioning	−0.40	0.79	−0.39	0.83	−0.18	0.66	0.28	0.43	0.25	0.41	0.32	0.34
Maximum span Digit Span backwards WISC/WAIS	3.79	1.12	4.00	1.22	4.41	1.31	4.25	1.20	4.60	1.23	4.89	1.29
Absolute deviation of median production Motor Timing Task (*ms*)	96.34	90.94	102.44	106.64	98.62	84.87	80.53	72.89	74.38	66.44	66.44	58.55
Percentage of deviation Time Test (*ms*)	21.46	14.32	19.85	16.46	13.77	8.63	14.09	8.85	12.90	7.33	11.12	5.94
SDRT^a^ ANT Baseline Speed (*ms*)	0.36	0.23	0.35	0.23	0.32	0.21	0.30	0.17	0.28	0.15	0.25	0.13
SDRT^a^ Motor Timing Task (*ms*)	0.37	0.29	0.35	0.33	0.27	0.20	0.22	0.08	0.20	0.07	0.18	0.05
RT ANT Baseline Speed (*ms*)	361.70	87.33	374.26	111.25	353.10	84.83	268.97	38.08	269.06	41.33	260.38	34.28
Mean absolute deviation ANT Tracking Task (*mm*)	2.93	2.09	2.34	1.56	1.99	1.60	2.56	1.48	2.35	1.14	2.15	1.15
Total IQ WISC/WAIS	95.44	14.85	101.73	12.97	105.59	13.21	94.77	16.27	100.96	15.17	106.72	14.19

Groups (ADHD/C, unaffected siblings and controls) were defined at baseline

*ADHD*, Attention-Deficit/Hyperactivity Disorder; *ANT*, Amsterdamse Neuropsychologische Taken; *CPRS-R:L*, Conners’ Parent Rating Scale-Revised: Long Version; *K-GAS*, Kiddie-Global Assessment Score; *RT*, Reaction time; *SDRT*, Standard deviation of reaction time; *SDQ*, Strengths and Difficulties Questionnaire; *WAIS*, Wechsler Adult Intelligence Scale; *WISC*, Wechsler Intelligence Scale for Children

^a^Corrected for mean reaction time

### Procedure

Testing at baseline and follow-up took place at the Vrije Universiteit Amsterdam, or at the Donders Institute in Nijmegen, the Netherlands. Participants were ≥ 48 h off medication during both baseline and follow-up assessments allowing complete wash-out (Greenhill et al. 2002). All ratings of behavioral functioning pertained to the participant’s functioning off medication. Families were financially compensated for participation. Informed consent was signed by all participants at both measurements, and parents signed for all children in their family as well. Ethical approval was obtained (CMO Regio Arnhem-Nijmegen; 2008/163; ABR: NL23894.091.08).

### Statistical Analysis

#### The Course of Neurocognitive Functioning

A linear mixed model was used to compare the course of neurocognitive functioning over the six year follow-up period of (1a) affected siblings (AS) versus controls, and (1b) unaffected siblings (US) versus controls. For all neurocognitive measures, we tested a group-by-time (baseline to follow-up) interaction-effect using a full-factorial model. In all models, family and subject were tested as random effects to account for within family correlation and for correlated measurements over time. Group was used as a fixed factor, and time as a repeated measure. Significance thresholds were set at 0.05. Please see for specific details regarding data preparation Supplement 1.

#### Covariates

In all analyses age at baseline and follow-up interval were included as covariates by default. Further, it was checked whether sex, pharmacological treatment, study site and the group-by-follow-up interval interaction effect confounded our findings, by adding these variables to the significant models.

#### Moderating Effects of Age

To explore potentially moderating effects of age, for all significant group-by-time interaction effects we tested the group-by-time (baseline to follow-up)-by-age (at baseline) interactions with a similar analytic procedure as described above. When significant, we retested the group-by-time effect in three equal sized age groups, to explore at which age catch-up has taken place.

#### Predicting ADHD Outcome from Neurocognitive Change

To investigate whether the course of neurocognitive functioning was related to ADHD outcomes (ADHD symptom severity and overall functioning) in affected and unaffected siblings, linear mixed models were used to account for familial dependence, with family as random effect. Change in neurocognitive performance between follow-up and baseline was calculated for each neurocognitive measure separately and used as fixed predictor in the analyses, while follow-up measures of ADHD symptom severity and overall functioning were used as outcome measures. For the time production measure, the statistical analysis differed slightly compared to that of the other variables, see Supplement 1.

#### Covariates

As there is a strong relationship between baseline and follow-up measures of symptom severity and impairment respectively, the baseline measures were fixed covariates in the respective mixed model-analyses. Age at baseline and follow-up interval were included as covariates by default. Further, when there were significant moderating effects between change in neurocognitive performance and age (or group; affected, unaffected siblings) on ADHD outcomes, we added the significant interaction effect to the main analysis investigating the relationship between neurocognitive change and outcome measures to account for differential age or group effects. Further, for our main analyses it was checked whether sex, pharmacological treatment, and study site confounded our results, by adding these variables to the significant models.

#### Sensitivity Analyses

As the results may have been impacted by including children with an IQ < 80, we checked whether results of our main analyses were robust when tested in a sample of children with an IQ ≥ 80.

## Results

Data quality check revealed that for included participants, 0.0%–1.4% of data per measure needed to be excluded mainly as a result of extreme outliers. As linear mixed models were used, these missing data points were taken into account by way of maximum likelihood estimation.

### The Course of Neurocognitive Functioning

**Fig. 1:**
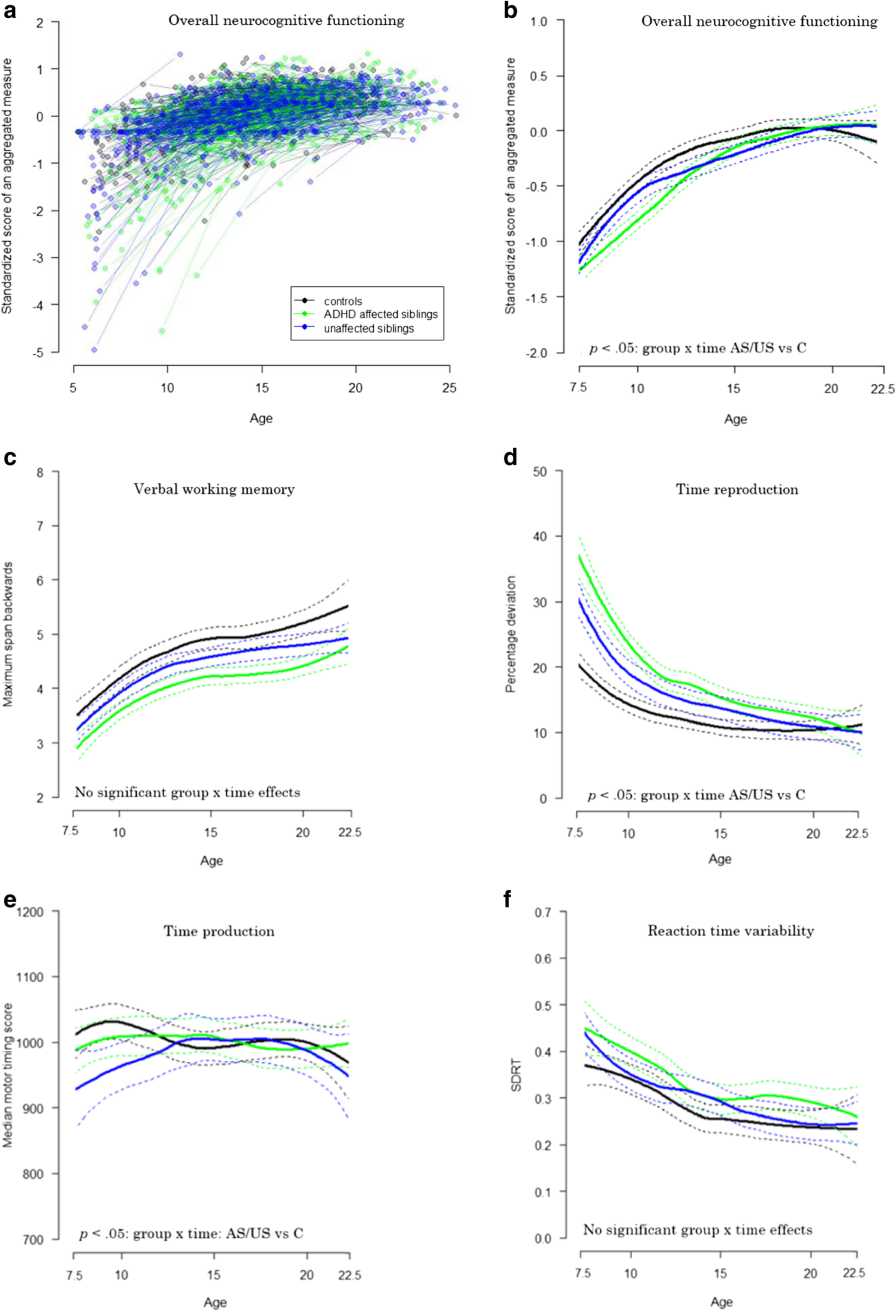

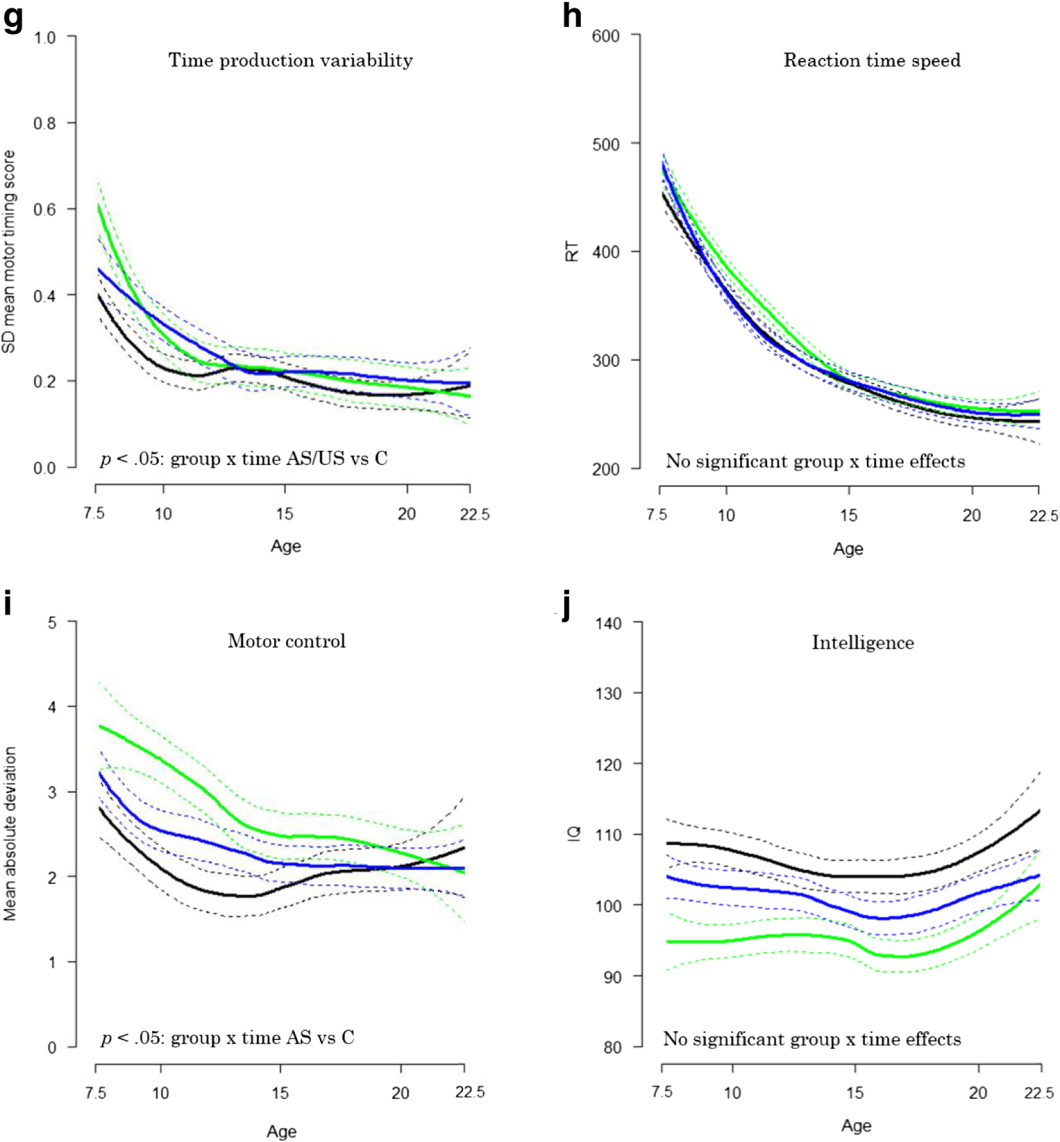
**a** Individual slopes of the overall measure of neurocognitive functioning over two timepoints (mean follow-up interval 6.0 years), as a function of age. **b-j** Estimated plots (Loess curve) based on the individual slopes (as an example plotted in Fig. 1a, for the overall measure of neurocognitive functioning). Dotted lines represent the 95% confidence interval. ADHD = Attention-Deficit/Hyperactivity Disorder; C = Controls; RT **=** Reaction time; SD = SD in ms, divided by the mean reaction time; SDRT = SD of mean reaction time in ms, divided by the mean reaction time; US = unaffected siblings

Table 2 displays the group-by-time interaction effects on neurocognitive development from baseline to follow-up, as well as the main effects of group. Figure 1a, b show the estimated plots for our aggregated measure of overall neurocognitive functioning, based on the individual slopes. Figure 1a illustrates that for overall neurocognitive functioning US and AS show a pattern of catch-up compared to controls (group x time interaction for AS vs controls: *b* = −0.18, *p =* 0.005*;* and US vs controls: *b* = −0.15, *p =* 0.024). Analysis on single measures of neurocognitive functioning also showed this pattern of catch-up indicated by a significant group x time interaction for time production (US vs controls: *b* = −0.28, *p =* 0.048), time reproduction (AS vs controls: *b* = −0.44, *p* < 0.001; US vs controls: *b* = −0.38, *p* = 0.001), time production variability (AS vs controls: *b* = −0.27, *p =* 0.01; US vs controls: *b* = −0.26, *p =* 0.03), and motor control (AS vs controls: *b* = −0.34, *p* = 0.008). For time production, unaffected siblings showed a stable pattern of slight underestimation of the 1000 ms interval over time, while controls showed overestimation of the 1000 ms interval at baseline and reached the level of (underestimation of) unaffected siblings at follow-up. For the remaining neurocognitive functions (US and AS: verbal working memory, reaction time variability, reaction time speed, intelligence; AS only: time production; US only: motor control), the group x time interaction was not significant. Post hoc analysis showed that for overall neurocognitive functioning, time production and motor control, there was no significant main effect of diagnostic group at follow-up (all *p*-values *>*0*.*093), indicating that full catch-up had taken place. For the other measures, a main effect of diagnostic group was found (all *p-*values <0.002; time production variability US vs controls: *d* = 0.46, AS vs controls: *d* = 0.67; time reproduction US vs controls: *d* = 0.30, AS vs controls: *d* = 0.39), indicating that although affected and/or unaffected siblings showed a pattern of catch-up and trended to performance levels of controls, for these measures, performance levels remained behind the level of controls at follow-up.

**Table 2:** Summary of statistical parameters in ADHD affected and unaffected siblings compared to controls

Neurocognitive domain	Parameter^a^	ADHD affected siblings vs controls			Unaffected siblings vs controls			All groups		
		*b*	*SE*	*p*	*b*	*SE*	*p*	*F*	*p*	Contrasts^b^
Overall neurocognitive functioning	Group x time	−0.18	0.06	**0.01**	−0.15	0.07	**0.02**			
	Group							2.063	0.16	
Verbal working memory	Group x time	0.01	0.10	0.95	−0.12	0.11	0.25			
	Group							20.135	**< 0.001**	0 > 1 > 2
Time production	Group x time	−0.21	0.12	0.09	−0.28	0.14	**0.046**			
	Group^c^							4.333	**0.01**	0 = 1,2; 1 > 2
Time reproduction	Group x time	−0.44	0.10	**< 0.001**	−0.38	0.11	**0**.**001**			
	Group^d^							12.122	**< 0.001**	0 > 1 > 2
Reaction time variability	Group x time	0.05	0.12	0.67	0.001	0.13	0.96			
	Group^e^							8.163	**< 0.001**	0 > 1 > 2
Time production variability	Group x time	−0.27	0.11	**0.01**	−0.26	0.12	**0.03**			
	Group							28.663	**< 0.001**	0 > 1 > 2
Reaction time speed	Group x time	−0.002	0.08	0.98	−0.13	0.10	0.19			
	Group							6.115	**0.002**	0 > 1 = 2
Motor control	Group x time	−0.34	0.12	**0.01**	−0.10	0.12	0.40			
	Group^f^							1.217	0.30	
Intelligence	Group x time	0.12	0.07	0.10	0.14	0.07	0**.**054			
	Group							27.194	**< 0.001**	0 > 1 > 2

Symbols that were provided in bold indicate a *p*-value < 0.05

0, Controls; 1, Unaffected siblings; 2, Affected siblings; *ADHD*, Attention-Deficit/Hyperactivity Disorder

^a^ Group x time interaction effects and main effects of group at follow-up were tested in separate analyses. ^b^ Based on mirrored z-scores, higher scores indicate better performance. ^c^ Sex and studysite were added as additional relevant confounders. ^d^ Sex was added as an additional relevant confounder. ^e^ Studysite was added as an additional relevant confounder. ^f^ Sex and pharmacological treatment were added as additional relevant confounders

#### Covariates

Analysis of the possible confounders revealed that sex significantly impacted on the two-way interaction model of time reproduction comparing unaffected siblings with controls (*p* = 0.003), however, when including sex in the model, the group x time interaction effect remained significant (*p* = 0.001). Group interacted significantly with follow-up interval in the two-way interaction model of time production comparing unaffected siblings with controls (*p* = 0.028), however, when including the group x follow-up interval interaction effect in the model, the group x time interaction remained significant (*p* = 0.048). None of the other potentially confounding relationships were significant (*p-*values between 0.13 and 0.99).

#### Moderating Effects of Age

**Fig. 2:**
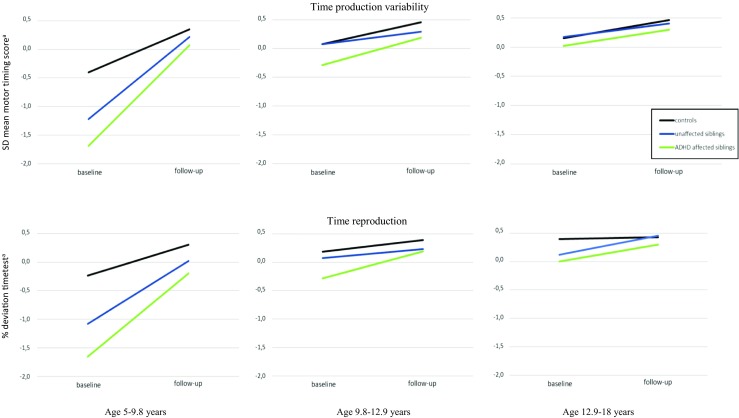
The course of time production variability and time reproduction (two timepoints, mean follow-up interval 6.0 years), separated for three age groups. SD = SD in ms, divided by the mean reaction time. ^a^ Based on mirrored z-scores, higher scores indicate better performance

To explore possible moderating effects of age on the course of neurocognitive functioning for the three diagnostic groups, group x time x age interactions were tested. The group x time x age interaction was significant for time production variability (AS vs controls: *b* = 0.13, *p* < 0.001; US vs controls: *b* = 0.11, *p* = 0.002), and time reproduction (AS vs controls: *b* = 0.07, *p =* 0.03). See Figs. 1 and 2 for visual plots. Further analysis in three equal sized age groups (ages 5–9.8 years; ages 9.8–12.9 years; ages 12.9–19 years) revealed that for time production variability (both AS and US) and time reproduction (AS), the group x time interaction effect was significant only in the youngest age group (*p* < 0.007).

### Predicting ADHD Outcome from Neurocognitive Change

Table 3 displays results of the relationship between neurocognitive change and both ADHD symptom severity and impairment at follow-up, over and above baseline ADHD symptom severity and impairment, respectively. Time production (higher score, less time-underproduction) at follow-up, adjusted for time production at baseline, was related to higher overall functioning at follow-up (*b* = 0.15, *p* = 0.001). No further significant relationships between neurocognitive change and either symptom severity or overall functioning at follow-up were observed (all *p-*values >0.07).

**Table 3:** Summary of statistical parameters regarding the relationship between neurocognitive change and ADHD outcomes

	Outcome					
	ADHD symptom severity			Overall functioning		
Predictor (standardized change scores)	*b*	*SE*	*p*	*b*	*SE*	*p*
Overall neurocognitive functioning	−0.05	0.04	0.22	−0.04	0.04	0.92
Verbal working memory	0.02	0.03	0.50	0.04	0.04	0.23
Time production (follow-up score)^a^	−0.02	0.04	0.55	0.15	0.04	0**.001**
Time reproduction^b^	−0.03	0.03	0.29	0.06	0.04	0.92
Reaction time variability	−0.002	0.03	0.94	−0.02	0.03	0.48
Time production variability	−0.03	0.03	0.22	−0.01	0.03	0.66
Reaction time speed	−0.03	0.05	0.50	−0.03	0.05	0.55
Motor control	0.01	0.03	0.68	−0.06	0.03	0.07
Intelligence	0.002	0.003	0.94	0.001	0.003	0.80

All models are adjusted for baseline age, follow-up interval and baseline symptom severity/parent reported impairment respectively. ADHD = Attention-Deficit/Hyperactivity Disorder

^a^Baseline time reproduction score were included in the models. ^b^ The interaction between group and time reproduction was included in the model for overall functioning

#### Covariates

Group interacted significantly with the predictive effects of change in time reproduction on overall functioning (*p* = 0.04) and therefore was taken into account as a covariate in all analyses on time reproduction and overall functioning. Both group (all *p-*values >0.13) and age (all *p-*values >0.06) did not significantly interact with neurocognitive change, indicating that relationships between neurocognitive change and outcomes were neither dependent on group (affected, unaffected siblings) nor on age. Analysis of the other possible confounders revealed that pharmacological treatment significantly impacted on the model of time production (*p* = 0.023), leaving the significant relationship between time production and overall functioning intact (*p* = 0.003). None of the other potentially confounding relationships were significant (*p-*values between 0.054 and 0.84).

### Sensitivity Analyses

Findings on the main analyses regarding the course of neurocognitive functioning and regarding the prediction of ADHD outcome using neurocognitive change replicated when participants with an IQ < 80 (*n* = 19) were excluded from the analyses. Similar or comparable (non-)significance levels and effect sizes were obtained.

## Discussion

Despite the central role of neurocognitive impairment in etiological models of ADHD, little is known about the longitudinal course of these neurocognitive characteristics and their relationship with outcomes of ADHD symptoms and overall functioning. The current study is the first to report on this in ADHD affected (*n* = 339) and unaffected siblings (*n* = 271), and controls (*n* = 228) in childhood/adolescence. In summary, over time, approximately half of the neurocognitive functions in affected and unaffected siblings trended near the level of controls (aggregated measure of overall neurocognitive functioning, time production- and reproduction, time production variability, reaction time speed, and/or motor control). However, within this trending pattern, only for time production, motor control, and overall neurocognitive functioning full catch-up took place, while for the other functions, there still was some difference in performance at follow-up between the groups. For verbal working memory, reaction time variability, and intelligence, the initial gap between performance of affected and unaffected siblings with controls remained stable over a 6-year period showing similar improvement over time. Importantly, in general, the course of neurocognitive functioning was not related to ADHD outcomes over and above baseline symptom severity or overall functioning in (un)affected siblings, suggesting that improvement/ deterioration of neurocognitive performance does not translate one-to-one into (ADHD) behavior.

In first instance, our finding that for approximately half of our measures both affected and unaffected siblings trended to the level of controls at follow-up, seems to fit the maturational lag theory. This process of normalization is in line with several previous studies that measured inhibitory control (Drechsler et al. 2005), response variability (using the stop-task; McAuley et al. 2014), a global measure of executive functioning, and attention (Miller et al. 2013). However, our findings indicate that for some functions that show a trend towards normalization, ADHD siblings still lag behind controls. Possibly, our sample may have been too young to show full catch-up. However, studies in adults show otherwise (Hervey et al. 2004; Mostert et al. 2015). Another puzzling part is that following the maturational delay hypothesis, catch-up of (un)affected siblings with levels of controls should be seen at a specific age, i.e. a specific age-related ‘growth spurth’, probably in late childhood/early adolescence with its major changes in brain development that parallel cognitive maturation (Giedd et al. 1999). However, only in three out of eighteen comparisons, trending of neurocognitive functioning in the direction of controls was dependent on age showing that the catch-up started already in (younger) childhood, with unaffected siblings reaching the level of controls somewhat earlier than affected siblings. Taken together, findings show greater complexity than expected based on the maturational delay hypothesis.

Notably, verbal working memory, reaction time variability, and intelligence did not show a trend into the direction of performance levels of controls at all, neither in affected nor in unaffected siblings. This is consistent with other studies on verbal working memory, reaction time variability, and intelligence (Biederman et al. 2009, 2008; Drechsler et al. 2005; McAuley et al. 2014; Miller et al. 2012, 2013; Vaughn et al. 2011), and confirmed in adults by meta-analyses (Hervey et al. 2004; Mostert et al. 2015). Indeed, numerous studies show that these neurocognitive impairments are key in ADHD (Castellanos and Tannock 2002; Martinussen et al. 2005; Tamm et al. 2012) and our finding that these functions remain impaired over time strengthens the proposed key role. In addition, findings do not show an evident pattern regarding to the type of measure that remains impaired or normalizes, as both ‘motor’ (reaction time variability) and ‘cognitive’ functions (verbal working memory, intelligence) were found to remain impaired or (partially) caught up. Clearly, our results emphasize there is no simple relationship between neurocognitive development and ADHD outcomes, and also suggest that ADHD is characterized by more than a maturational lag in neurocognitive functioning.

The lack of association between the course of neurocognitive functioning and ADHD outcomes, even without correction for multiple testing, is consistent with studies showing that the course of different types of neurocognitive functions from childhood to young adulthood is largely independent of current diagnostic status or ADHD symptom change (Coghill et al. 2014a, b; Drechsler et al. 2005; McAuley et al. 2014; Miller et al. 2012; van Lieshout et al. 2013). Studies that showed (at least some) positive relation between neurocognitive improvement and ADHD outcomes differed from our study by investigating a preschool sample (Rajendran et al. 2013a, b); by studying different cognitive processes (e.g. delayed matching to sample; Coghill et al. 2014a, b); or by investigating girls only (Miller et al. 2013). Regarding our positive finding for time production, we should take into account the possibility of a type I error. However, it is possible that time production ability may be a relevant measure for every day functioning, such as planning (Allman and Meck 2012), which may impact, for example, on the ability to be ready on time, to cook, or to evaluate the feasibility of a certain time schedule. If so, this measure may more directly relate to overall functioning than to symptoms of ADHD itself. Taken together, it may be concluded that the relation between neurocognition and expression of the ADHD phenotype over time is not as straightforward as was commonly thought.

The remarkable absence of a relationship between change in neurocognitive functioning and symptoms of ADHD, neither in affected nor their unaffected siblings leads us to suggest this best fits a model in which neurocognitive deficits are not directly related to ADHD symptoms, i.e. do not lie in the causative chain, as was commonly thought. Our findings thus might best fit an epiphenomenal model, in which neurocognitive dysfunctions in ADHD are seen as some form of comorbid condition, perhaps relating to the same underpinnings as ADHD symptoms, but not mediating this relationship (Coghill et al. 2014a; van der van der Meer et al. 2013). This is in line with other studies that for example showed that persistent genetic factors underlie the longitudinal relationship between ADHD and intelligence in twins (Rommel et al. 2015), or found shared genetic etiology between several neurocognitive functions (e.g. memory, reaction time speed, reasoning abilities), and psychiatric symptoms (Hagenaars et al. 2016).

However, it may be premature to firmly conclude that neurocognitive functions are not (at all) causally related to the disorder. For example, the domain of executive functioning includes more functions beside verbal working memory, e.g. visuo-spatial working memory, inhibitory control, set shifting. Also, other executive functioning paradigms (for example tasks with greater trial numbers, or tasks placing greater demand on central executive functioning) may have been better able to measure verbal working memory abilities compared to the Digit Span task used in the current study (Kasper et al. 2012; Tarle et al. 2017). Further, there are neurocognitive functions that have not been measured in the current study, such as motivation or reward related neurocognitive functions. Nevertheless, the results in this large sample are very consistent regarding the absent relationship between neurocognitive functioning and ADHD outcomes. Although we consider it unlikely that adding one or two domains or changing the type of measures will lead to convincing and strong relationships between neurocognitive functioning and ADHD outcomes, further study is needed to support or nuance the current conclusion. Related to this point is that it has been demonstrated that methodological variability may explicate inconsistencies in findings (see for example Alderson et al. 2013 for factors that may explain inconsistencies regarding the relationship between working memory and ADHD). Therefore, we think it may be important to further explore other approaches. For example, a person-based approach may reveal new insights, acknowledging the complex interplay (e.g. strengths and weaknesses) between neurocognitive functions within one individual as well as neurocognitive heterogeneity that may exist in the ADHD population; such studies are not often performed yet (see for example Bergwerff et al. 2017; Fair et al. 2012; Rommelse et al. 2016). In addition, recent studies have shown the validity of a general continuous psychopathology factor (so-called ‘P factor’) as an alternative approach for the DSM-based classifications of mental disorders. Possibly, this dimensional cross-cutting of psychopathology may be a valuable transdiagnostic approach (Caspi et al. 2014; Martel et al. 2016), that may increase the value of neurocognitive functions on predicting behavior beyond the narrow-defined DSM-based categories or symptoms of ADHD, thereby acknowledging commonly existent comorbidities. Another hypothesis, based on studies showing that neurocognitive functioning is related to ADHD outcome especially in younger children (Pauli-Pott and Becker 2011; Rajendran et al. 2013a, b; Sjöwall et al. 2015; van Lieshout et al. 2013), is that neurocognitive functions may be involved in the onset of ADHD, but not in the further course of ADHD (e.g. persistence/remittance). Perhaps in younger years, neurodevelopmental factors have a larger impact (for example it may be possible that greater brain plasticity at a younger age leads to more forceful compensating mechanisms for negative environmental and/or biological [e.g. injury] impact), while during development environmental factors may play an increasingly greater role (e.g. parenting styles, peer relationships, school performance/failure, self-esteem; Sonuga-Barke and Halperin 2010). This may suggest that remittance of ADHD is far more difficult to predict and may be impacted by many more and other variables compared to the early onset of ADHD.

The results should be viewed in the light of some strengths and limitations. As outlined above, this is the first study to investigate the course of neurocognitive functioning in relation to ADHD outcomes at two timepoints, including several neurocognitive functions and continuous outcome measures - containing overall functioning as well -, thereby taking into account the role of age and pharmacological treatment, in a large sample. In terms of limitations, some aspects of our sample limit generalization to the (ADHD) population, including our exclusive focus on individuals with the combined type of ADHD (Lara et al. 2009), the limited representation of girls in our sample – although models did not change when taking sex into account -, and the inclusion of only Caucasian participants. For reasons of feasibility, we included single measures of multiple neurocognitive domains instead of using multiple measures of one single domain, which would have increased reliability of our measurements of the neurocognitive domains. However, in line with our current findings, we did not find strong and convincing relationships between neurocognitive functioning and ADHD outcomes in an earlier study that used multiple assessments for one neurocognitive construct (van Lieshout et al. 2017), considering it unlikely that this may have impacted our results. Also, the use of a single item rating scale as an index of overall functioning is limited and may have precluded the possibility to detect meaningful relationships between neurocognitive functioning and specific domains of impairment, such as academic achievement. Further, we did not investigate the possible differential relationship between the two ADHD symptom axes (inattention versus hyperactivity/impulsivity) and neurocognitive functioning, since this was beyond the scope of our paper. However, this might be of relevance. For example, Rapport et al. (2009) have suggested that increased activity levels augment arousal needed for working memory performance in all children, and specifically in children with ADHD due to chronic cortical underarousal (Rapport et al. 2009). However, as the separate symptom axes are strongly interrelated and also strongly correlated to total symptom severity, and no clear relationship was found between neurocognitive functioning and ADHD total symptoms, it would be unexpected to find meaningful relationships between neurocognitive functions and specific symptom axes.

Taken together, the present study provides insight into the course of multiple neurocognitive domains in ADHD affected and unaffected siblings compared to controls, and studied how change in neurocognitive functioning is related to ADHD outcomes. Some neurocognitive functions trended in the direction of, or fully caught-up, with normative performance, while other important neurocognitive functions (i.e. verbal working memory, variability in responding) remained impaired while symptoms improved, and no clear association between neurocognitive change and ADHD outcomes was found at all. Our findings question the etiological link between neurocognitive deficits and ADHD.

## Electronic supplementary material

ESM 1 (DOCX 35 kb)
