# Supplementary material for: The Course of Neurocognitive Functioning and Prediction of Behavioral Outcome of ADHD Affected and Unaffected Siblings
Source: J Abnorm Child Psychol. 2018 Aug 6;47(3):405–19. doi: 10.1007/s10802-018-0449-z (PMC6397140; doi:10.1007/s10802-018-0449-z)
Supplement: Supplementary file 1 — (DOCX 35 kb) [file 10802_2018_449_MOESM1_ESM.docx]

**The Course of Neurocognitive Functioning and Prediction of Behavioral Outcome of ADHD Affected and Unaffected Siblings.**

**Supplement 1**

**Attrition Analyses**

Due to later implementation of the neurocognitive assessment in the baseline protocol for ADHD affected and unaffected siblings, 15.6% and 21.0%, respectively, had no baseline neurocognitive assessment. Some participants declined neurocognitive assessment at follow-up, and only filled out questionnaires, resulting in 7.9% of all participating children having no follow-up neurocognitive assessment. For other variables missing data was < 5%. Cases with missing data were taken into account as linear mixed modelling estimates missing data by way of maximum likelihood estimation.

Attrition was investigated on predictor (eight neurocognitive domains), behavioral (parent reported ADHD symptoms and impairment, oppositional behavior, anxiousness, social problems), and demographic variables (age, sex) at baseline, using Generalized Estimating Equation analyses (GEE). Attrition rates were not significantly different in the three groups (affected siblings, unaffected siblings, control children: *p* = .065). Across groups, participants lost to follow-up had lower IQ-scores at baseline (*p* = .002; 99.2 versus 102.8) than participants successfully followed-up. In the group with affected siblings, participants lost to follow-up had a lower number of ADHD total symptoms (*p* = .035; 33.7 versus 35.7) than participants successfully followed-up. In the group with unaffected siblings, participants lost to follow-up had lower IQ scores (*p* = .000; 96.7 versus 103.5) than participants successfully followed-up. In controls, participants lost to follow-up had better scores on timing reproduction (*p* = .020; 11.5% deviation versus 13.8% deviation) than participants successfully followed-up. Group comparisons between participants successfully followed-up with complete data and participants successfully followed-up but with neurocognitive measures available only at one measurement, revealed no significant differences on any of the neurocognitive, behavioral, and demographic variables (*p* > .079).

**Participant inclusion**

Between 2003 and 2006, the IMAGE study recruited families with at least one child with clinically diagnosed ADHD/C and at least one sibling regardless of possible ADHD status, and recruited control families with none of the family members having a history of ADHD. Clinical diagnosis of each participant with ADHD/C was assessed by health-care professionals from clinical child care centers in The Netherlands. In addition, the diagnosis was confirmed using an extensive assessment protocol described below.

Parent and teacher questionnaires were used to screen participants: Conners’ Parent Rating Scale-Revised: Long version (CPRS-R:L; Conners, Sitarenios, Parker, & Epstein, 1998) and Strengths and Difficulties Questionnaire (Goodman, 1997). *T*-scores ≥ 63 on the Conners DSM-IV ADHD subscales Inattention (L), Hyperactivity/impulsivity (M), and Total symptoms (N), and scores ≥ 90^th^ percentile on the SDQ Hyperactivity subscale were considered clinical (see for reference of classification levels for example Lezak, Howieson, Bigler, & Tranel, 2012). The cut-off scores were used as an indicator for further (comprehensive) diagnostic assessment, chosen to prevent exclusion of possible ADHD cases solely based on questionnaire data. Psychometric properties of the Conners’ questionnaire appear adequate, demonstrated by Cronbach’s α between .75 and .94, test-retest reliability for the externalizing subscales between .42 and .78, and effective discriminative power with sensitivity, specificity, positive and negative predictive power > 90% (Conners et al., 1998). Similarly, psychometric properties of the SDQ were satisfactory, demonstrated by generally independent internal and external scales, a mean Cronbach’s α of .73, and a retest stability of 0.62 (Goodman, 1992). Participants scoring clinically on any of these subscales were administered the Parental Account of Children’s Symptoms (PACS), a semi-structured, standardized, investigator-based interview with the parents as informants (Taylor, 1986). Original inter-rater agreement coefficients for the PACS were ranging from 0.79-0.96 (Chen & Taylor, 2006), internal consistency of the ADHD section was good (Cronbach’s alpha = 0.89) and concurrent validity of the PACS diagnosis was confirmed by point-biserial correlations with Conners’ Parent and Teacher rating scale N (0.78 and 0.68; Chen et al., 2008). Inter-rater agreement for the PACS in our own sample was 0.88 (range 0.71-1.00; Müller et al., 2011). Next to a specific ADHD section, comorbid conditions such as emotional problems (anxiety, depression), disruptive behavior (oppositional defiant behavior and conduct disorder), comorbid disorders (such as tic disorders, substance misuse disorders, obsessive compulsive disorders) and autistic symptoms (children that were known to have a diagnosis of autism were excluded on forehand) were investigated. With that information, it could be more carefully assessed whether ADHD symptoms arise from ADHD and were not the result of another disorder. This was done, for example, by explicitly investigating whether symptoms of inattention, hyperactivity and/or impulsivity also existed in periods without or with fewer symptoms arising from other mental disorders (such as mood or anxiety disorders). For participants using medication, ratings were gathered of participants’ functioning off medication (for example during weekends, holidays, evenings – when medication effects could have worn off – and the days before the assessment in which the children were at least 48 hours off their medication).

A standardized algorithm was applied to the PACS to derive each of the 18 DSM-IV ADHD symptoms, providing precisely defined scoring rules that translate the several PACS items into the 18 specific behavioral ADHD symptoms. These were combined with items that were scored 2 (‘pretty much true’) or 3 (‘very much true’) in the teacher rated Conners’ ADHD subscales (L, M and N) to generate the total number of hyperactive-impulsive and inattentive symptoms of the DSM-IV symptom list. Symptoms of the CTRS-R:L were only added to the combined symptom count if at least two symptoms were reported to avoid the Conners’ score putting too much weight on the diagnosis. Situational pervasiveness was defined as some symptoms occurring in two or more different situations as indicated by the parents in the PACS interview, as well as the presence of at least one symptom scoring 2 or 3 from the ADHD subscales (L, M and N) as indicated by the teachers on the Conners’ questionnaire (Conners’ Teacher Rating Scale-Revised: Long version [CTRS-R:L], Rommelse et al., 2007a). For the follow-up assessment, a similar procedure was followed, but now either a teacher rating (Conners’ Teacher Rating Scale–Revised: Long version [CTRS-R:L] applied for children<18 years) or a self-report (Conners’ Adult ADHD Rating Scales–Self-Report: Long version [CAARS-S:L] applied for children ≥18 years, subscales E, F, and G) was used (von Rhein et al., 2015). Further, instead of the PACS interview, participants were administered the Dutch translation of the Schedule for Affective Disorders and Schizophrenia for School-Age Children–Present and Lifetime Version (K-SADS-PL: (Kaufman et al., 1997) containing developmentally appropriate questions to assess each of the 18 ADHD symptoms, compatible with DSM-IV-TR (American Psychiatric Association, 2000) as well as comorbid conditions. Reliability and validity tests were satisfying, with inter-rater reliability between 0.80 and 0.90, test-retest reliability in the fair to good range (*ĸ*=.52-.70) and convergent, divergent and discriminant validity showing significant (*p*<.05) moderate to excellent outcomes (Birmaher et al., 2009; Kaufman et al., 1997). Parents reporting on their children, and the children themselves, if ≥12 years old, were interviewed separately. Initially, all participants were administered the K-SADS ADHD screening interview. Participants with elevated scores on any of the screen items were administered the full ADHD supplement. Final scores on each item of the K-SADS were determined by weighing all available information.

Participants with a combined symptom count of at least six symptoms of hyperactive/impulsive behavior and/or inattentive behavior following the algorithm were diagnosed with ADHD, provided they: (a) met the DSM-IV criteria for pervasiveness and impact of the disorder (measures derived from the semi-structured interview), (b) showed an age of onset before 7 at baseline, which was extended to an age of 12 at follow-up following DSM-5 criteria, and (c) received a *T*-score ≥ 63 on at least one of three scales on at least one of the Conners’ ADHD questionnaires (i.e. CPRS-R:L and/or CTRS-R:L for children<18 years and CPRS-R:L and/or CAARS-S:L for children ≥18 years), pertaining to a period without medication. Participants with a combined symptom count of at least six symptoms who did not meet one or more of these criteria were evaluated by a team of trained experts to derive a consensus decision on their diagnosis. For young adults (≥18 years), criteria were adapted slightly such that a combined symptom count of ﬁve instead of six symptoms was sufﬁcient for a diagnosis (Kooij et al. 2005).

Participants with a diagnosis of ADHD/C based on the algorithm at baseline were included in the current study, as well as their unaffected siblings and controls that both did not meet criteria for ADHD at study entry, nor the first degree family members of controls. Subjects that participated at both baseline and follow-up were included in the current study, except participants that had no neurocognitive assessment either at baseline nor at follow-up (1.4%).

**Neurocognitive variables**

**Overall measure of neurocognitive functioning.** An aggregated measure containing several aspects of neurocognitive functioning may be more sensitive than specific aspects in isolation. Therefore, we calculated an overall measure of neurocognitive functioning using principal component analysis (PCA) on the correlation matrix containing intercorrelations between all eight neurocognitive measures obtained at baseline and detailed below (raw scores). Variables correlated < .53 with each other. The one-factor solution had an Eigenvalue of 2.70 and explained 33.69% of variance. To obtain an overall measure of neurocognitive functioning from this PCA at baseline and follow-up, component score coefficients derived from the PCA at baseline were multiplied with the z-scores on the eight neurocognitive measures at baseline and follow-up, respectively. These scores were summed, separately for the baseline and follow-up measures.

**Verbal working memory.** For verbal working memory, the maximum span of Digit Span Backwards (WISC-III and WAIS-III, depending on the child’s age; Wechsler 2000, 2002) was used. There is debate on whether this measure is a valid assessment of verbal working memory. The rationale for selecting this measure is that there is evidence that the Digit Span task activates brain areas commonly associated with working memory (Rossi et al., 2013), and it has shown to discriminate between children with ADHD (and their parents) and control children (and their parents; Rommelse, Altink, Oosterlaan, Buschgens et al., 2008; Thissen et al., 2014)*.* Validity was confirmed by a strong correlation of the Digit Span Backwards (*r* = 0.48) with a visuo-spatial working memory task (higher cognitive load; number of correctly identified targets in correct order) assessed at baseline in our ADHD affected sample.

**Timing.** For time production, accuracy on the Motor Timing Task was used. Accuracy was measured by median of time production in ms, which reflects under- versus overproduction (time production smaller or larger, respectively, than 1000ms; Van Meel et al., 2005; Rommelse, Altink, Oosterlaan, Beem, et al., 2008). We looked at trials in the neutral condition, which were the first forty trials of the task. For time reproduction, precision of time reproduction was used as measured with the Time Test Application Version 1.0. Precision was measured as the percentage of deviation between the response length and the stimulus length in the visual modality, averaged over four trials of the three longest intervals: 12, 16, and 20 seconds (Barkley, 1998; Rommelse et al., 2007a).

**Timing variability.** For reaction time variability, variability on the ANT (Amsterdamse Neuropsychologische Taken) Baseline Speed Task was used (performed with the left hand). Variability was measured by the SD of reaction times (ms) divided by the mean reaction time calculated for the 32 trials of the task (Rommelse, Altink, Oosterlaan, Beem et al., 2008). The ANT is a standardized computerized neurocognitive battery developed in The Netherlands (De Sonneville, 2014). For time production variability, variability on the Motor Timing Task was used. Variability was measured by the SD of mean reaction time in ms, divided by mean reaction time, on the first 40 trials of the task.

**Reaction time speed.** For reaction time speed, reaction time (in ms) on the 32 trials of the Baseline Speed Task, performed with the left hand, was used (Rommelse, Altink, Oosterlaan, Beem et al., 2008).

**Motor control.** For motor control, precision (mean absolute deviation in mm) on 60 equal parts of the circle of the Amsterdam Neuropsychological Tests (ANT) Tracking Task, performed with the left hand, was used (Rommelse et al., 2007b).

**Intelligence.** Total IQ was estimated by two subtests of the WISC-III or WAIS-III (depending on the child’s age; Wechsler, 2000, 2002): Vocabulary and Block Design. These subtests are known to correlate in the .90 range with full administration Full Scale IQ (Sattler, 2001, in Groth-Marnat, 2003).

**Statistical analysis**

In order to study neurocognitive development we have chosen to keep neurocognitive tasks similar at baseline and follow up and use questionnaires with similar informants for the entire age range, to prevent task effects and reporter bias. To ensure data quality, it was checked for each task and each participant whether task instructions were clear and additionally, data were checked for oddities, e.g. performance at chance levels, extreme within-group outliers (>4 SD), bottom or ceiling effects, and if necessary, data were excluded. There were no indications that for a certain task, structural problems in the youngest or eldest age groups occurred: e.g. visual inspection combined with values of skewness and kurtosis revealed normal variation in scores in the full sample, as well as in the 1/3 youngest participants and 1/3 eldest participants. In addition, it was checked during the assessment whether parents were able to confidently fill in the questionnaires; if not (e.g. parents and participants hardly had any contact), that questionnaire was excluded from our dataset. In two earlier studies, we have demonstrated that using parents as informants in older children did not affect the findings (blind review). Raw scores of the CRS questionnaires (with age taken into account in the models tested) were analyzed as the standardized scores of the CPRS are only available for children until the age of 18.

**References**

American Psychiatric Association (2000). *Diagnostic and statistical manual of mental disorders: DSM-IV-TR*: American Psychiatric Publishing, Inc.

Barkley, R. A. (1998). Time perception application (version 1.0 software). University of Massachusetts Medical Center/Chesapeake Technology, Boston.

Birmaher, B., Ehmann, M., Axelson, D.A., Goldstein, B.I., Monk, K., Kalas, C., . . ., Brent, D.A. (2009). Schedule for affective disorders and schizophrenia for school-age children (K-SADS-PL) for the assessment of preschool children – A preliminary psychometric study. *Journal of Psychiatric Reseach, 43,* 680-686.

Chen, W., & Taylor, E., (2006). Parental Account of Children’s Symptoms (PACS), ADHD phenotypes and its application to molecular genetic studies. In R.D. Oades, (Ed), *Attention‐deficit hyperactivity disorder (ADHD) and the hyperkinetic syndrome (HKS): Current ideas and ways forward* (pp. 3–20). Hauppauge, NY: Nova Science Publisher.

Chen, W., & the Image Consortium (2008). DSM‐IV combined type ADHD shows familial association with sibling trait scores: A sampling strategy for QTL linkage. *American Journal of Medical Genetics, Part B: Neuropsychiatric Genetics*, *147B*, 1450–1460.

Conners, C. K., Sitarenios, G., Parker, J. D. A., & Epstein, J. N. (1998). The revised Conners' Parent Rating Scale (CPRS-R): factor structure, reliability, and criterion validity. *Journal of abnormal child psychology, 26*(4), 257-268.

De Sonneville, L. M. J. (1999). Amsterdam Neuropsychological Tasks: a computer-aided assessment

program. In: BPLM den Brinker, PJ Beek, AN Brand, SJ Maarse, & LJM Mulder (Eds), Cognitive ergonomics, clinical assessment and computer-assisted learning: Computers in Psychology, Vol 6. Lisse, Swets & Zeitlinger, ISBN 9026515537, pp 187-203.

De Sonneville, L. M. J. (2014). Handboek ANT Amsterdamse Neuropsychologische Taken. Amsterdam, Boom Test Uitgevers, ISBN 9789461059932.

Goodman, R. (1997). The Strengths and Difficulties Questionnaire: a research note. *Journal of Child Psychology and Psychiatry, 38*(5), 581-586.

Goodman, R. (2001). Psychometric properties of the Strengths and Difficulties Questionnaire. *Journal of the American Academy of Child and Adolescent Psychiatry, 40*(11), 1337-1345.

Groth-Marnat, G. (2003). *Handbook of psychological assessment, 4th ed.* Hoboken, NJ: John Wiley & Sons, Inc.

Kaufman, J., Birmaher, B., Brent, D., Rao, U., Flynn, C., Moreci, P., . . . Ryan, N. (1997). Schedule for Affective Disorders and Schizophrenia for School-Age Children-Present and Lifetime Version (K-SADS-PL): initial reliability and validity data. *Journal of the American Academy of Child and Adolescent Psychiatry, 36*(7), 980-988.

Lezak, M. D., Howieson, D. B., Bigler, E.D., Tranel, D., 2012. *Neuropsychological Assessment.* New York, NY: Oxford University Press, Inc.

Müller, U. C., Asherson, P., Banaschewski, T., Buitelaar, J. K., Ebstein, R. P., Eisenberg, J., . . . Steinhausen, H.C. (2011). The impact of study design and diagnostic approach in a large multi-centre ADHD study. Part 1: ADHD symptom patterns. *BMC Psychiatry, 11*, 54.

Rommelse, N. N. J., Altink, M. E., Oosterlaan, J., Beem, L., Buschgens, C. J. M., Buitelaar, J., & Sergeant, J. A. (2008). Speed, variability, and timing of motor output in ADHD: Which measures are useful for endophenotypic research? *Behavior Genetics, 38*(2), 121-132.

Rommelse, N. N., Altink, M. E., Oosterlaan, J., Buschgens, C. J., Buitelaar, J., & Sergeant, J. A. (2008). Support for an independent familial segregation of executive and intelligence endophenotypes in ADHD families. *Psychological Medicine, 38*(11), 1595-1606

Rommelse, N. N. J., Altink, M. E., Oosterlaan, J., Buschgens, C. J. M., Buitelaar, J., De Sonneville, L. M. J., & Sergeant, J. A. (2007b). Motor control in children with ADHD and non-affected siblings: deficits most pronounced using the left hand. *Journal of Child Psychology and Psychiatry, 48*(11), 1071-1079.

Rommelse, N. N. J., Oosterlaan, J., Buitelaar, J., Faraone, S. V., & Sergeant, J. A. (2007a). Time reproduction in children with ADHD and their nonaffected siblings. *Journal of the American Academy of Child and Adolescent Psychiatry, 46*(5), 582-590.

Rossi, S., Lubin, A., Simon, G., Lanoe, C., Poirel, N., Cachia, A., et al. (2013). Structural brain correlates of executive engagement in working memory: children's inter-individual differences are reflected in the anterior insular cortex. *Neuropsychologia, 51*(7), 1145-1150.

Taylor, E. A. (1986). Childhood hyperactivity. *British Journal of Psychiatry, 149*, 562-573.

Thissen, A. J. A. M., Rommelse, N. N. J., Hoekstra, P. J., Hartman, C., Heslenfeld, D., Luman, M., . . .. Buitelaar, J. B. (2014). Attention deficit hyperacitivty disorder (ADHD) and executive functioning in affected and unaffected adolescents and their parents: Challenging the endophenotype construct. *Psychological Medicine, 44*, 881-892.

Van Meel, C. S., Oosterlaan, J., Heslenfeld, D. J., & Sergeant, J. A. (2005). Motivational effects on motor timing in attention-deficit/hyperactivity disorder. *Journal of the American Academy of Child and Adolescent Psychiatry, 44*(5), 451-460.

von Rhein, D., Mennes, M., van Ewijk, H., Groenman, A. P., Zwiers, M. P., Oosterlaan, J., . . . Buitelaar, J. (2015). The NeuroIMAGE study: a prospective phenotypic, cognitive, genetic and MRI study in children with attention-deficit/hyperactivity disorder. Design and descriptives. *Europeand Child and Adolescent Psychiatry, 24*(3), 265-281.

Wechsler, D. (2000). *WAIS-III Nederlandstalige Bewerking Technische Handleiding.* The Psychological Corporation: London.

Wechsler, D. (2002). *WISC-III Handleiding.* The Psychological Corporation: London.

Supplemental Table 1

*Description of Instruments*

| Task | Measure | Description | References |
| --- | --- | --- | --- |
| WISC/WAIS-III Digit Span task | - Maximum Span backwards | An auditory task to measure the accuracy of verbal working memory; a sequence of numbers was announced, and should be replicated backwards, with increasing length. | Wechsler, 2000/2002 |
| Motor Timing Task | - Median of time production (ms)  - SDRT, corrected for MRT (ms) | A computerized task to measure the accuracy and variability of motor timing, using the first 40 neutral trials of the task, requiring a response on a button when a subject thought a 1-second interval had elapsed after a tone. | Rommelse et al., 2008  Van Meel, Oosterlaan, Heslenfeld, & Sergeant, 2005 |
| Time Test | - Percentage of deviation visual modality (mean of three highest intervals) | A computerized task to measure the precision of the reproduction of five time intervals (4, 8, 12, 16, 20sec); A light bulb (visual modality) was presented for a specific interval length, which had to be reproduced thereafter. | Rommelse et al., 2007a  Barkley, 1998 |
| ANT Baseline Speed | - SDRT, corrected for MRT left hand  - RT (ms) left hand | A computerized task to measure the speed and variability of motor output in response to an external cue (simple reaction time task). | Rommelse et al., 2008  De Sonneville, 1999 |
|  |  |  |  |
| ANT Tracking | - Absolute deviation left hand | A computerized task to measure the precision of motor control; an invisible midline should be traced with a mouse cursor as quickly and precisely as possible, between an inner and an outer circle. | Rommelse et al., 2007b  De Sonneville, 1999 |
| WISC/WAIS-III vocabulary, block design | - Estimated total IQ | Two subtests of the WISC/WAIS were used to estimate full-scale IQ. | Wechsler 2000/2002 |

**ANT** Amsterdamse Neuropsychologische Taken **MRT** Mean reaction time **RT** Reaction time **SDRT** Standard deviation of reaction time **WAIS** Wechsler Adult Intelligence Scale **WISC** Wechsler Intelligence Scale for Children.
